# Integrative Transcriptomic and Metabolomic Analyses Elucidate the Molecular Mechanisms Underlying Enhanced Yield and Bacterial Blight Resistance in the RXN2 Rice Cultivar

**DOI:** 10.3390/plants14182921

**Published:** 2025-09-19

**Authors:** Ji’an Bi, Jingqi Wang, Xuan Huang, Jiefeng Jiang, Xianbo Shi, Genliang Bao, Qiufeng Meng, Chengqi Yan

**Affiliations:** Institute of Crop Research, Ningbo Academy of Agricultural Sciences, Ningbo 315040, China

**Keywords:** growth–defense trade-off, phytohormone, transcriptomic, metabolomic, grain yield, bacterial blight

## Abstract

Achieving high yield while maintaining disease resistance is a crucial goal in rice breeding programs. In this research, two cultivated rice varieties, Jia58 and Runxiang3, were selected as parental lines. A new variety, designated as the new variety RXN2, was generated and identified through a breeding process that involved hybridization of the parental lines followed by irradiation-induced mutagenesis of the offspring. Compared with its parental lines, RXN2 shows increased plant height, higher yield, and stronger resistance to bacterial blight. Comprehensive transcriptomic and metabolic analyses indicate that pathways associated with growth, such as gibberellin and auxin signaling, are upregulated in RXN2. Meanwhile, defense-related pathways, especially those involving jasmonic acid and peroxidase metabolism, are significantly enhanced. These results provide new insights into the trade-offs between growth and defense and elucidate the genetic and metabolic underpinnings of the simultaneous improvement in grain yield and disease resistance in rice.

## 1. Introduction

Rice (*Oryza sativa* L.) is a crucial staple crop globally and has a significant impact on worldwide food security [1,2,3]. The increasing global population and the impacts of climate change have heightened the challenges for rice production, thereby necessitating a deeper understanding of the mechanisms that regulate the simultaneous enhancement of yield and stress resilience [1,4].

Radiation-induced mutagenesis serves as a powerful tool to swiftly augment genetic diversity, a crucial factor for breeding advancements [5]. By employing gamma ray-induced mutation breeding, the mutants derived from Samundchini, Vishnubhog and Jhilli manifested significantly reduced plant height, accelerated time to maturity, and superior yield performance relative to their respective parental lines [6]. Moshen et al. utilized gamma rays as a mutagen to enhance oil and protein contents alongside high yield traits in soybean [7]. In the context of irradiating susceptible pea varieties, namely Shijiadacaiwan 1 (SJ1) and Chengwan 8 (CW8), nine SJ1 and five CW8 mutants displayed resistance to *Fusarium wilt*. Remarkably, the same five CW8 mutants also showed resistance to powdery mildew [8].

Crossbreeding leverages hybrid vigor (heterosis) to enhance both yield and stress tolerance [9,10]. The exploitation of hybrid vigor, where hybrid offspring outperform their parental lines in traits such as biomass and grain yield, has transformed rice cultivation [11]. Recent genomic studies have revealed that the establishment of hybrid dominance is not merely determined by dominant or super-dominant genetic mechanisms, but is also associated with the enhancement of energy metabolic networks [12,13]. For instance, the activation of the TOR complex in the hybrid rice Shanyou 63 (SY63) significantly improves energy utilization efficiency and promotes panicle development and yield enhancement by regulating glycolysis and tricarboxylic acid cycle metabolism [14]. Moreover, the dominant complementary effects of disease resistance genes (e.g., *Xa21*, *RPM1*) exhibit synergistic resistance in hybrid progeny, highlighting the significance of multigene interactions in hybrid dominance [15]. These findings underscore the necessity to incorporate metabolic and epigenetic regulation into models of hybrid dominance for precision breeding. However, the molecular basis of hybridization is intricate, involving multi-gene interactions, epigenetic regulation, and environmental interactions, which remain to be fully elucidated. Meanwhile, the dynamic balance between growth and resistance in rice is crucial for its adaptive capacity, and this process may be dually regulated by hormone signaling networks and metabolite accumulation.

Phytohormones, as core signaling molecules, play a pivotal role in regulating plant growth and development and stress response [16,17,18]. Auxin, brassinosteroids (BR), and gibberellin (GA) directly promote vegetative growth by regulating cell division and elongation, while salicylic acid (SA) and jasmonic acid (JA) dominate the signal transmission of stress response [19]. GA signaling affects rice plant height, seed germination, and flowering time through a DELLA protein-mediated regulatory network [20]. The semi-dwarf rice varieties that were widely used in the Green Revolution relied on loss-of-function mutations in the *SD1* gene (encoding a key enzyme for GA synthesis) [21]. Auxin/IAA and auxin response factor (ARF) are important components in the growth hormone signaling pathway. When growth auxin levels are elevated, growth hormone induces the degradation of Aux/IAA proteins through the 26S ubiquitylation pathway and releases ARF proteins, which in turn activate the regulation of auxin-responsive genes by ARF, and it has been shown that the interplay of OsIAA3 with the OsARF16 influence the expression of *OsBUL1*, a positive regulator of seed size, through interactions [22]. JA and SA are the main defense-related hormones in response to invading pathogens [23]. JA has been widely demonstrated to enhance resistance against herbivory and pathogen infection [24]. JA and its derivatives promote rice resistance to *Xanthomonas oryzae* pv. *oryzae* (*Xoo*), the causal agent of bacterial leaf blight, a major global rice disease [25,26,27].

Plant hormones synergistically or antagonistically regulate the balance between plant growth and stress resistance [17,18]. The OsBZR1–SPX1/2 module integrates JA and BR to balance plant growth and fungal blats resistance in response to Pi availability [28,29]. Abscisic acid (ABA) antagonizes JA signaling and reactive oxygen species (ROS) to negatively regulate rice virus infection [30]. JA signaling regulates insect pest inhibition of rice growth by promoting GA metabolism, and JA modulates the GA metabolism enzymes GA2ox3 and GA2ox7, which can convert active GA to inactive GA, leading to growth inhibition [31]. It has been reported that *OsGRF6* regulates the expression of genes involved in the auxin and JA signaling pathway by targeting downstream genes, *OsYUCCA1* and *OsWRKY82*, thereby enhancing both rice yield and bacterial blight resistance [32]. GA-ABA antagonism dynamically balances growth and drought tolerance, with elevated ABA closing stomata to conserve water but inhibiting GA-driven cell expansion. By precisely regulating GA levels, both alkalinity resistance and grain yield were simultaneously achieved in rice, breaking the negative correlation between stress tolerance and growth in traditional breeding [33].

The peroxidase (Prx) gene family is involved in pathogen resistance in rice. *Xoo* enhances infection by inducing the expression of *OsPrx30* to scavenge H_2_O_2_ levels and reduce ROS accumulation [34]. Overexpressing *OsPrx26* enhances rice resistance to *Xoo,* whereas *OsJMTF1,* a transcription factor in the JA signaling pathway, enhances antimicrobial properties by activating the expression of *OsPrx26* [35]. Similarly, knockout of *OsPrx4* or *OsPrx13* enhance resistance to *Xoo* and *M. oryzae* infection [36]. Furthermore, OsBZR1 directly binds to the promoter of the *OsPrxs* to inhibit the expression levels of *OsPrxs* and ROS, leading to BR-mediated susceptibility to pathogen infection [37].

In recent years, the development of metabolomics and transcriptomics technologies has provided new perspectives for analyzing the synergistic mechanisms of growth and resistance in rice. Primary metabolites, such as sugars and amino acids, have a direct impact on the efficiency of biomass accumulation. In contrast, secondary metabolites, including phenols and terpenoids, enhance defense through mechanisms like increasing cell wall toughness or regulating ROS homeostasis. Moreover, they work in synergy with hormonal signaling to bolster defense mechanisms without compromising growth [14].

In this work, two rice cultivars from Zhejiang, China, namely Jia58 (J58) with high yield potential but relatively weaker disease resistance, and Runxiang3 (RX3) with lower yield but stronger resistance, were intercrossed, and the resulting hybrid progeny were subjected to gamma irradiation. Through rigorous selection, a progeny line, designated as RXN2, was identified. RXN2 displays enhanced resistance to *Xoo* with greater plant height and higher grain yield compared to both parental cultivars. Integrated analysis of the transcriptome and metabolome revealed that the RXN2 variety accomplished high yield and disease resistance through the activation of pathways such as GA, auxin, JA and Prx. These findings shed new light on the complex growth–defense trade-offs, offering valuable insights into the genetic and metabolic basis of breeding for promoting both disease resistance and yield.

## 2. Results

### 2.1. RXN2 Displayed Higher Grain Yield and Enhanced Bacterial Blight Resistance

**Figure 1:**
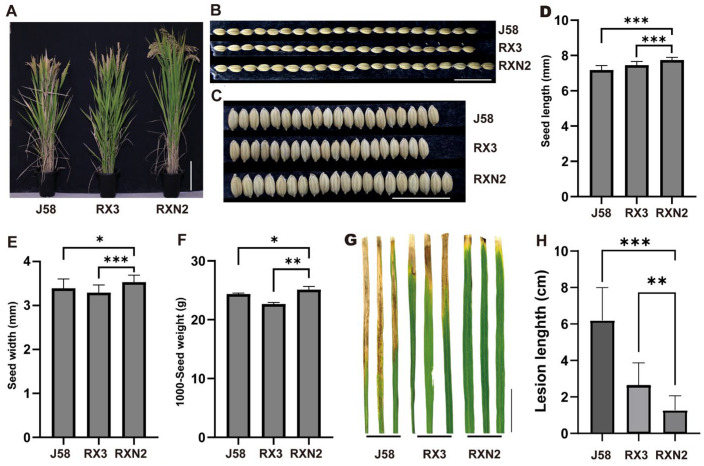
Yield-related traits and bacterial resistance of J58, RX3 and RXN2 plants. (**A**–**C**) Phenotypes of mature plants, seed length and seed width. Scale bar = 20 cm for (**A**), 2 cm for (**B**,**C**). (**D**–**F**) Comparison of seed length, seed width and 1000-seed weight. (**G**) Bacterial blight symptoms in J58, RX3 and RXN2 leaves at 14 days after inoculation with *Xoo*. Bar = 2 cm. (**H**) Lesion length on the fifth leaf blades at 14 days after inoculation with *Xoo*. Values are mean ± s.d from three biological replicates., Student’s *t*-test, * <0.05, ** <0.01, *** <0.001; *n* = 20 for (**D**,**E**), *n* = 3 for (**F**) and *n* = 21 for (**H**).

To develop a high-yielding and disease-resistant rice variety, we crossed the cultivated rice varieties J58 with RX3, followed by gamma-ray irradiation of the hybrid progeny seeds. Through screening, we identified a superior plant, which was designated as RXN2. We observed that the RXN2 plants were significantly taller than J58 and RX3 (Figure 1A). In addition, the seed length and seed width of RXN2 were greater than those of J58 and RX3 (Figure 1B–E). Consistently, RXN2 displayed higher 1000-seed weight compared with RX3 and J58 (Figure 1F). Furthermore, when inoculated with *Xoo*, the mean lesion lengths in J58, RX3 and RXN2 were 6.19 cm, 2.63 cm and 1.27 cm, respectively (Figure 1G,H), indicating that RXN2 was more resistant to *Xoo* infection than its parents. Together, RXN2 demonstrated higher grain yield with enhanced resistance.

### 2.2. Transcriptome Analysis

To investigate the regulated genes related to agronomic traits, a high-throughput sequencing approach was employed to compare the expression profiles among J58, RX3 and RXN2. This yielded 402 million raw reads, totaling 60.633 Gb. After quality filtering, each cDNA library produced 40.144–47.83 million clean reads, summing to 59.071 Gb, with an average of 6.563 Gb per library. Quality metrics showed that over 96% of bases had Phred scores ≥ 30, ranging from 96.06% to 96.27%. The high-quality clean reads exhibited balanced base compositions with GC contents above 44%, indicating excellent sequencing quality suitable for downstream analyses (Table S1).

**Figure 2:**
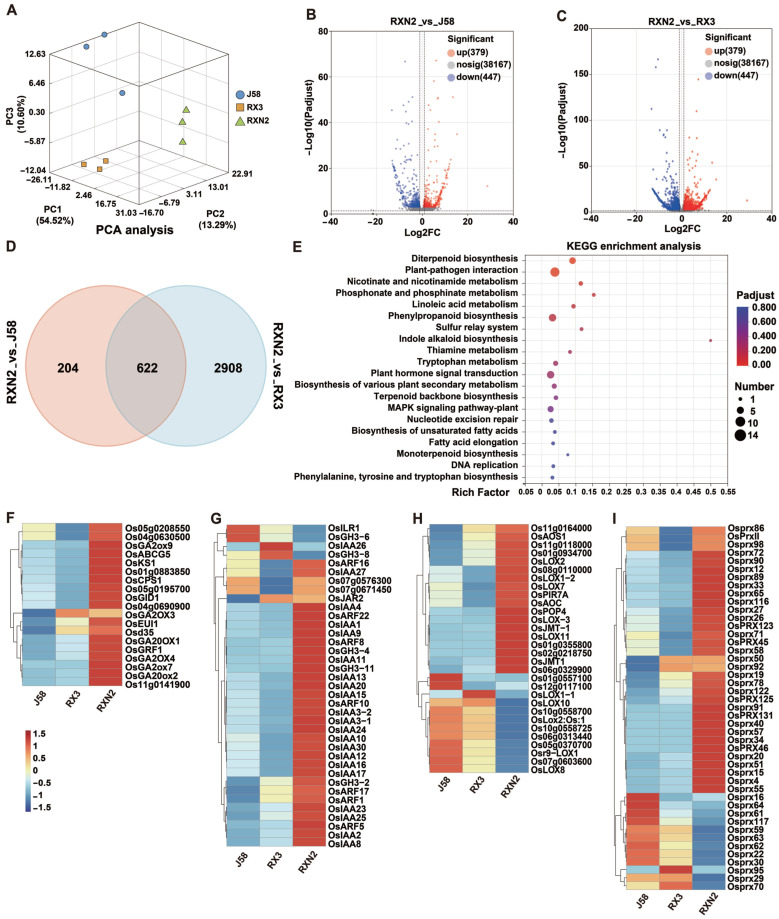
Transcriptome analysis of differentially expressed genes. (**A**) Sample Principal Component Analysis (PCA). The two mutually perpendicular coordinates at the bottom indicate principal component 1 and 2, respectively, and the vertical coordinates indicate principal component 3. Each variety contains three replicates consisting of at least ten seedlings. (**B**,**C**) Volcano plots for the RXN2_vs_J58 and RXN2_vs_RX3 comparison groups. Red indicates up-regulated genes, blue indicates down-regulated genes, and grey indicates non-DEGs. (**D**,**E**) Venn and KEGG analysis of DEGs in (**B**,**C**). (**F**,**I**) Heatmap of gene expressions associated with GA (**F**), auxin (**G**), JA (**H**) and Prx (**I**).

**Figure 3:**
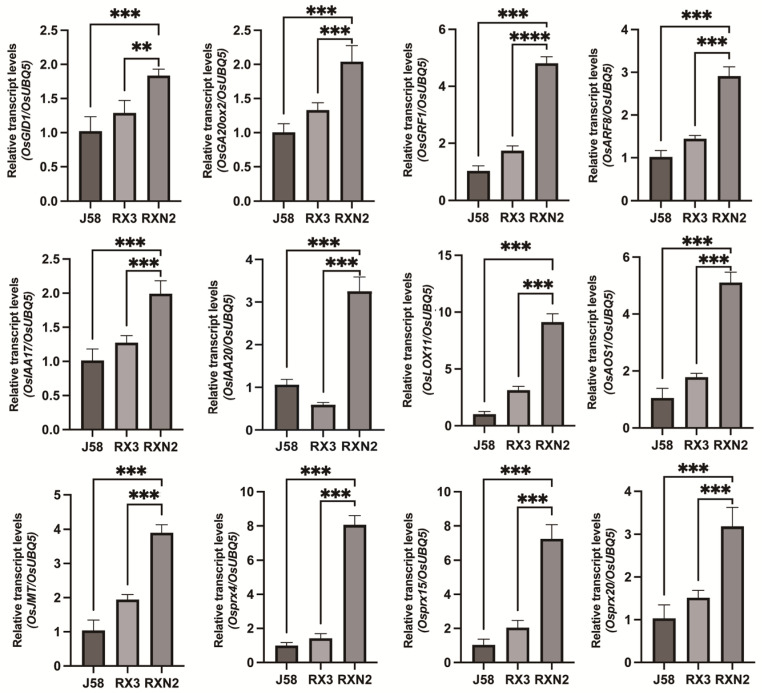
RT-qPCR verification of the DEGs in J58, RX3 and RXN2. The *OsUBQ5* was used as negative control. Three independent biological repeats were performed. Values are mean ± s.d from three biological replicates., Student’s-*t* test, ** <0.01, *** <0.001, **** <0.0001, *n* = 3.

The PCA indicated that the data was reproducible and of high quality (Figure 2A). Based on the |log_2_(fold change, FC)| ≥ 1 and FDR < 0.05 screening threshold, a total of 3734 DEGs were identified, including 826 DEGs (379 up-regulated and 447 down-regulated) in the comparator group RXN2_vs_J58, while 2530 DEGs (2404 up-regulated and 1126 down-regulated) in the comparator group RXN2_vs_RX3 (Figure 2B,C, Table S2). Venn analysis indicated that a total of 622 genes were altered in RXN2 compared with both J58 and RX3 (Figure 2D). The enrichment of altered genes was further analyzed in the Kyoto Encyclopedia of Genes and Genomes (KEGG)database, and the top 20 pathways with the highest enrichment levels were listed (Figure 2E, Table S3). Pathways related to growth, such as Plant hormone signal transduction (ko04075) and Cyanoamino acid metabolism (ko00460), and related to defense, including Plant–pathogen interaction (ko04626), Linoleic acid metabolism (ko00591), MAPK signaling pathway (ko04016) were up-regulated. Our further hierarchical clustering analysis showed that genes related to growth-promoting hormones, GA and Auxin, and related to defense signaling, JA and Prx, were significantly upregulated in RXN2 plants (Figure 2F–I). Subsequently, we conducted RT-qPCR to detect the expression levels of the altered genes. As shown in Figure 3, The GA-related genes (*OsGRF1*, *OsGID1*, and *OsGA20ox2*), Auxin-related genes (*OsARF8*, *OsIAA17*, and *OsIAA20*), JA-related genes (*OsLOX11*, *OsAOS1*, and *OsJMT*), and PRXs (*OsPRX4*, *OsPrx15*, and *OsPrx20*) in RXN2 plants were higher than those in J58 and RX3. These results implied that the GA, Auxin, JA, and Prx pathways were activated in RXN2 plants.

### 2.3. Metabolomics Analysis

**Figure 4:**
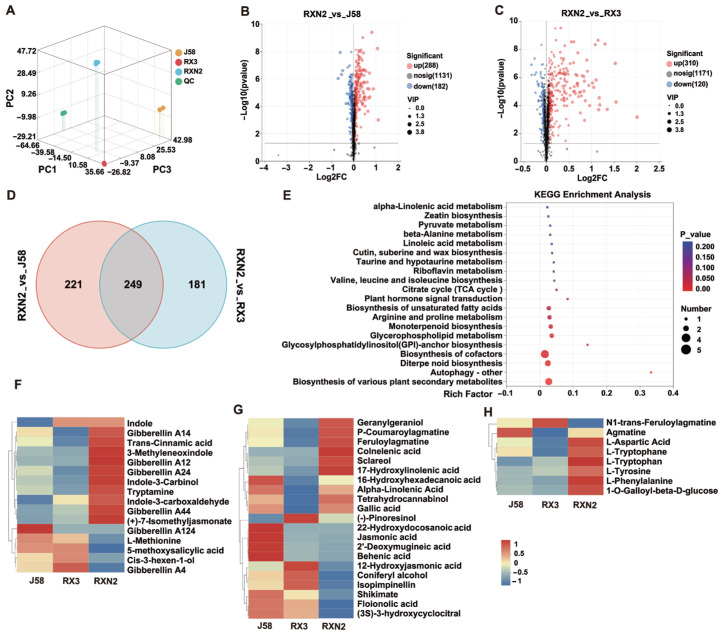
Metabolome analysis of. (**A**) Sample Principal Component Analysis (PCA). Each variety contains three replicates consisting of at least ten seedlings. (**B**,**C**) Volcano plots for the RXN2_vs_J58 and RXN2_vs_RX3 comparison groups. (**D**,**E**) Venn and KEGG analysis for DEMs in (**B**,**C**). (**F**–**H**) Heat map of metabolite levels associated with plant yield (**F**), disease resistance (**G**) and both plant growth and defense (**H**).

To obtain an overview of the metabolite changes, a widely targeted metabolomic analysis was carried out among J58, RX3 and RXN2. We observed that the three replicates of each combination type are very close to each other by PCA, indicating that the data are reproducible and consistently high quality (Figure 4A). Based on the metabolite annotations and screening thresholds (*p* < 0.05 and VIP > 1) in the Metabolite Information Database, we identified a total of 651 differential metabolites (DEMs) (Figure 4B,C, Table S5). There were 470 DEMs (288 up and 182 down) in the comparison group RXN2_vs_J58, whereas 430 DEMs (120 up and 310 down) in the comparison group RXN2_vs_RX3, respectively, (Figure 4B,C). To further analyze the DEMs, a VENN analysis was performed. The results showed that a total of 249 DEMs were commonly identified in both groups (Figure 4D). Based on the enrichment of KEGG pathways, the DEMs are mainly concentrated in pathways related to plant hormone signal transduction, which is associated with growth and development, as well as α-linolenic acid metabolism, cutin, suberine, and wax biosynthesis and diterpenoid biosynthesis, and biosynthesis of diverse plant secondary metabolites (Figure 4E, Table S5).

Further hierarchical clustering analysis revealed many metabolites are related to rice growth and development; these metabolites belonged to the phytohormone family, including GA, indole compound, and their derivatives (e.g., indole-3-carbinol), whose levels were significantly elevated in the hybrid progeny (Figure 4F). Compared with J58 and RX3, The RXN2 exhibited higher level of GA12, GA14, GA24, GA44, and GA124, whereas lower GA4 (Figure 4F and Table S4). Aside GAs, other terpenoid such as isocyperol, steviol, (S)-(-)-Perillyl alcohol and ineketone were enriched (Table S4). The DFMs related to plant defense were also enriched (Figure 4G). Colneleic acid is the key node and a substrate for the synthesis of defense-related metabolites in the LOX pathway, and **α**-linolenic acid is the precursor of JA biosynthesis. We observed that α-linolenic acid and 16-Hydroxylinolenic acid belonging to α-linolenic acid metabolism were significantly more prominent in RXN2 than in J58 and RX3 (Figure 4G and Table S4). Furthermore, metabolites involved in phenylpropanoid metabolism such as paeonoside and Coniferyl were also enriched in The RXN2 (Table S4). In addition, alcohol Sclareol and geranylgeraniol, the precursors for phytochemical synthesis, were significantly increased in RXN2 plants. P-Coumaroylagmatine, the defense-oriented phenylamine derivative, was also present in the hybrid progeny at higher levels. Furthermore, growth and defense-related amino acid and their derivatives, such as L-tryptophan, L-phenylalanine and L-tyrosine were also enriched in RXN2 (Figure 4H). Together, these data indicate that there is a significant elevation in the synthesis and accumulation of growth and development-related hormones and defense-related secondary metabolites contributing to the high yield and enhanced resistance phenotype of RXN2.

### 2.4. Joint Analysis of the Transcriptome and Metabolome

**Figure 5:**
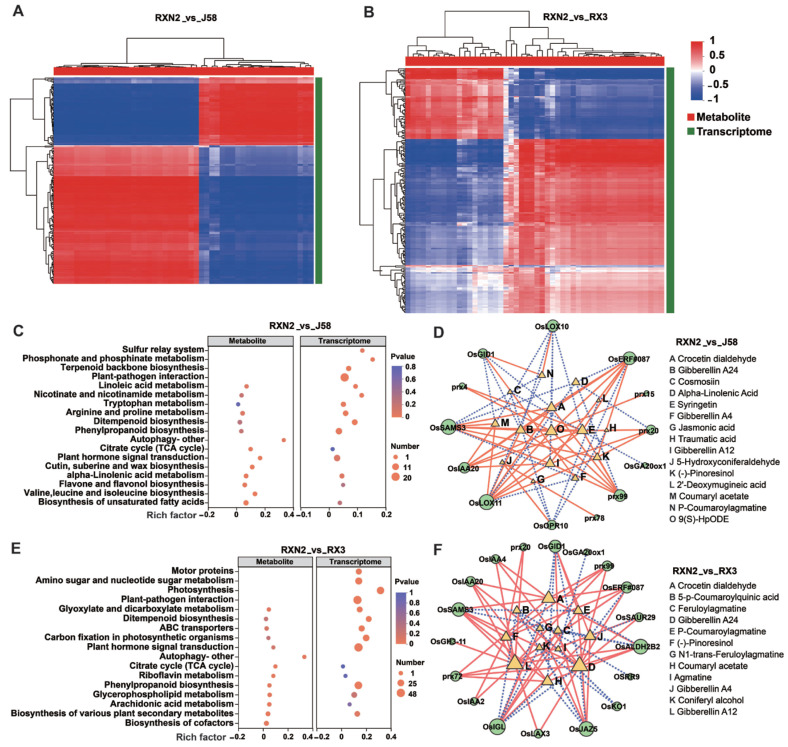
Correlation analysis of transcriptomic and metabolomic data. (**A**,**B**) Heatmap of expression correlation between DEGs and DEMs. Each row in the figure represents a gene and each column represents a metabolite. Each grid in the figure represents the correlation between a gene and a metabolite. (**C**,**E**) Bubble diagram of KEGG enrichment of (DEGs and DEMs. The boxes on the left are metabolites, the right are genes, and the number of metabolites and genes is indicated by the size of the dots. (**D**,**F**) Correlation network diagram for DEGs and DEMs. Green circles represent genes and yellow triangles represent metabolites, with specific names of metabolites labelled on the right side of the network diagram; the size of the circles and triangles indicates the magnitude of expression. The red solid line presents positive correlation, and the blue dashed line is negative correlation.

To further explore the mechanisms underlying the high yield and resistance traits in RXN2, we performed a combined transcriptomics and metabolomics analysis. The expression correlation heatmap generated using Pearson’s correlation algorithm showed that many genes were positively correlated with metabolites (Figure 5A,B), suggesting that these genes may directly or indirectly regulate the accumulation and changes in the corresponding metabolites). Subsequently, we performed KEGG pathway enrichment analyses on the transcriptome and metabolome. Pathways such as diterpenoid biosynthesis, plant hormone signal transduction, plant–pathogen interaction and phenylpropanoid biosynthesis were significantly enriched in RXN2 (Figure 5C,E). To elucidate the intermolecular interactions between differential metabolites and differential genes in these metabolic pathways, the top 100 relationship pairs were selected and analyzed using Pearson’s correlation coefficient. We observed that the genes involved in phytohormone signaling pathways and diterpenoid biosynthesis were positively correlated with metabolites in both the RXN2_vs_J58 and RXN2_vs_RX3 comparator groups (Figure 5D,F). In the RXN2_vs_J58 comparison group, *OsLOX11* was negatively correlated with JA biosynthesis precursor α-linolenic acid (Figure 4D). In the RXN2_vs_RX3 comparator group, *OsPrx20*, *OsPrx99*, and *OsIAA2* were positively correlated with metabolites belonging to (-)- pinoresinol (Figure 4C,D). In the both RXN2_vs_J58 and RXN2_vs_RX3 comparator groups, *OsSAMS3*, *OsPrx20*, and *OsERF#087* were positively correlated with GA12 and GA24, whereas *OsGID1* was negatively correlated with GA24 (Figure 5D,F). Collectively, these results suggest that the metabolites in these pathways are directly or indirectly regulated by their corresponding differential genes.

## 3. Discussion

In the process of rice breeding, hybridization offers great possibilities for the enhancement of its genetic diversity and adaptive capacity, which is essential for rice to improve its competitiveness in the market [38]. Using small wild rice (BBCC genome) as the donor and the indica rice variety IR24 as the acceptor, the introgression lines (ILs) are developed through backcrossing and selection. Phenotypic evaluation showed changes in morphology and yield-related traits. Notably, ILs 41, 11, and 7 exhibited high resistance to leaf blight [39]. It has also been shown that four rice hybrids, ILH299, ILH326, ILH867, and ILH901, have significant advantages in grain yield when compared to their parents [40]. In this study, two rice varieties, J58 with higher yield potential, whereas RX3 with better resistance were hybridized and then exposed to radiation mutagenesis, resulting in the successful development of a new rice hybrid, RXN2, with both yield and defense superior to its two parents.

The growth-promoting hormones, GA and Auxin, play an important role in plant growth and development [40]. Exogenous application of GA enhances crop yield [41]. GA20ox is responsible for the conversion of GA9 and GA20 to GA12 and GA53, and mutation of *OsGAox2* produces rice dwarf varieties [42]. Overexpression of *OsGID1*, the GA receptor, resulting in taller plants than wild type [43,44]. *OsGRF1*, involved in GA-induced stem elongation, positively regulates grain size [45,46]. In this work, transcriptome data revealed that many genes involved in the GA pathway were up-regulated in RXN2 plants, and RT-qPCR demonstrated higher expression level of *OsGID1*, *OsGA20ox2*, and *OsGRF1* (Figure 2 and Figure 3). Consistently, RXN2 exhibited higher concentration of GA_12_ and GA_24_ but lower level of GA_4_ (Figure 4). This profile is typical of a negative feedback response in which elevated GA20-oxidase and/or GA2-oxidase activities convert active GA_4_ into inactive forms, thereby lowering endogenous GA_4_ levels and, consequently, plant height [47]. This is also related to the fact that the rice used for metabolomic analysis was 30-day-old seedlings; previous reports show that rice accumulates large amounts of inactive GAs before stem elongation, which are then rapidly converted into active GAs during the tillering and jointing stages [48,49]. These together indicate that GA signaling contributes to the growth and yield-related agronomic traits of RXN2. The plant hormone auxin, indole-3-acetic acid (IAA), also plays a pivotal role in plant growth [19]. ARF transcription factors are positive regulators whereas auxin-responsive protein IAAs serve as repressors in auxin signaling. OsARF16 antagonistically interacts with OsIAA3 to regulate seed size and yield [22]. The OsIAA10-OsGSK5-OsARF4 module activates the growth hormone signaling pathway, leading to increased grain size and thousand-grain weight through the modulation of glume cell expansion [22]. In this work, compared with J58 and RX3, higher transcript levels of auxin-related genes were observed and confirmed by transcriptome and RT-qPCR in RXN2 plants (Figure 2 and Figure 3). Furthermore, the metabolomics data showed that many indole compounds were also enriched in RXN2 plants (Figure 3). These results suggest that auxin signaling was involved in the formation of larger grain size and higher yield in RXN2.

JA and its derivatives modulate plant defense against pests and pathogen infections [19]. The enzymes of LOX and AOS catalyze JA biosynthesis and plays a role in pathogen resistance [50,51].Overexpression of *OsLOX9* enhances rice resistance to fungal blast [50]. In addition, OsJMTF1, a jasmonate-responsive MYB transcription factor, plays a positive role in the rice defense against *Xoo* infection [34]. In this work, we observed the activation of JA signaling in RXN2 (Figure 2, Figure 3, Figure 4 and Figure 5), indicating that the JA pathway was involved in enhanced *Xoo* resistance in RXN2. Class III Prx (EC1.11.1.7) are induced after pathogen infection and can enhance plant defense [35]. Enhanced expressions of *OsPrx11*, *OsPrx13*, *OsPrx16*, *OsPrx28*, and *OsPrx65* contribute to rice against viral attacks [35]. Overexpression of *OsPrx26* enhances rice resistance to *Xoo* [34]. In our work, many *OsPrx* genes exhibited higher transcriptional levels in the hybrid progeny RXN2 (Figure 2). RT-qPCR demonstrated higher expression of *OsPrx4*, *OsPrx15*, and *OsPrx20* genes (Figure 5). Thus, our data reveals the involvement of Prx signaling in regulating bacterial defense in RXN2.

Increasing studies show that the metabolism of α-linolenic acid, terpenoid, and phenylpropanoid plays a crucial role in plant defense mechanisms. Transcriptomic and metabolomic analyses have revealed that the metabolic pathway is upregulated in the leaves of the disease-resistant cotton variety Zhongzhimian 2, while it is downregulated in the leaves of the susceptible variety Xinluzao 7 [52]. Activation of α-linolenic acid enhances JA-mediated defense in various plants [53,54]. Overexpression of *OsTPS19*, the terpene synthase gene that functions as an (S)-limonene synthase, enhances rice resistance to the blast fungus *M. oryzae* [55]. *OsTPS24*, which encodes a jasmonate-responsive monoterpene synthase that produces the antibacterial compound γ-terpinene, promoting rice resistance against *Xoo* infection [56], highlighting the importance of terpenoid metabolism in enhancing plant defense. In addition, through mediating redirection of the phenylpropanoid pathway, pathogen-induced glycosyltransferase (UGT73C7) enhances *Arabidopsis* disease resistance to *Pseudomonas syringae pv.* tomato *DC30000* [57]. Consistently, our data also revealed that the α-linolenic acid, terpenoid, and phenylpropanoid metabolic pathways in RXN2 are activated, contributing to its enhanced resistance to bacterial blight compared to its parent strain (Figure 2, Figure 3 and Figure 4).

There are trade-offs between resistance and growth [58]. Cross-talk between growth hormones (auxin, BR, cytokinin (CK) and GA) and defense hormones (JA, SA and ethylene) serves as an important mechanism underlying growth–defense trade-offs [50]. Overexpression of *OsBZR1* enhances fungal resistance but results in the dwarf phenotype of rice [59]. BRs antagonistically cross-talk with salicylic acid SA and GA pathways to regulate root elongation and oomycete *Pythium graminicola* resistance [60]. Herein, we found that simultaneous activation of GA, auxin and JA signaling in RXN2, leads to taller plants and higher yield with enhanced bacterial resistance compared with J58 and RX3, providing new insights into the understanding of growth–defense trade-offs.

## 4. Materials and Methods

### 4.1. Plant Material and Growing Conditions

Two *O. sativa* L. japonica cultivar J58 and RX3, along with their progeny RXN2 were used in this study. The rice variety RXN2 was developed through a combination of hybridization, radiation mutagenesis, and systematic selection. It originated from the cross between J58 and RX3. The F1 seeds derived from this hybridization were planted, and the resulting seeds (F2 generation) were treated with 200 Gy ^60^Co γ-ray radiation. After the mutagenesis, the treated seeds were sown to initiate the breeding program. Through six consecutive generations of rigorous selection based on phenotypic stability, agronomic performance, and genetic uniformity, the genetically stable variety RXN2 was successfully developed.

The germinated seeds were transferred to standard rice culture solutions and grown hydroponically in a greenhouse under a 12-h light (30 °C)/12-h dark (25 °C) photoperiod, with a photon density of 200 μmol m^−2^s^−1^ and approximately 60% humidity, as previously described [25,29]. One-month-old plants inoculated in the same tone were collected and immediately frozen in liquid nitrogen and stored at −80 °C for subsequent analysis.

To evaluate yield traits, the J58, RX3, and RXN2 plants were cultivated in the same paddy fields located in Ningbo City, Zhejiang Province, China, during the period from June to November 2024. The rice seeds were first soaked in water at 37 °C in the dark for two days to induce germination. Subsequently, the seedlings were grown in the same soil seedbed for four weeks prior to transplantation into the paddy fields. The yield was assessed based on the 1000-seed weight measured in the paddy fields.

### 4.2. Pathogen Inoculation

For the plant resistance assay, the *Xoo* strain PXO99 (P6) was employed as described [25]. Briefly, after an overnight incubation, bacterial cells were harvested, cleansed, and re-suspended. The concentration was then calibrated to 1 × 10^6^ colony-forming units (CFU)/mL in sterilized water. The fifth fully expanded leaf blades of rice plants served as the target for inoculation utilizing the clipping method. As a control, leaves were clipped using scissors dipped in sterilized water. Disease severity was assessed by measuring lesion length (in centimeters). Specifically, at 14 days post-inoculation (dpi), inoculated leaves were collected, and the distance from the leaf tip to the advancing edge of the grayish lesion was meticulously measured with a ruler. The ruler used had a maximum measurement capacity of 30 cm, with the smallest gradation being 1 mm. For each treatment, at least 10 plants were used, and 20 leaves were measured for average lesion length analysis, and the entire procedure was independently repeated three times.

### 4.3. RNA-Seq Library Preparation and Sequencing

Library construction and sequencing were performed by Majorbio (Shanghai, China). One-month-old rice shoots from J58, RX3, and RXN2 were collected, ground, and total RNA was extracted using the Trizol method. The Illumina^®^ Stranded mRNA Prep and Ligation methods were employed for library construction and sequencing. Differential expression analysis was conducted using the DESeq2 software version 1.42.0 (https://bioconductor.org/packages/release/bioc/html/DESeq2.html, accessed on 30 August 2023). Genes with an FDR < 0.05 and |log2FC| ≥ 1 were identified as significantly differentially expressed. These differentially expressed genes (DEGs) were subsequently analyzed and annotated in the KEGG pathway database (version 202309, http://www.genome.jp/kegg/, accessed on 12 September 2024). Three biological replicates for each cultivar were prepared, with each replicate consisting of pooled tissue from at least ten individual seedlings.

### 4.4. Total RNA Extraction and RT-qPCR

RT-qPCR was conducted to analyze the changes in mRNA levels as described [29]. One-month-old rice shoots were harvested, and total RNA was extracted using the LEYIBIO TRIzol protocol (LEYIBIO, Hangzhou, China), then the RNA was reverse transcribed to cDNA using the LEYIBIO First Strand cDNA Synthesis Kit (with dsDNase) (LEYIBIO, China). OsUBQ5 was selected as the internal reference gene. Quantification was performed using the SYBR Green I fluorescent dye method on a QuantStudio™ 6 Flex real-time fluorescent quantitative PCR instrument, following the instructions for preparation of the reaction system using the Hieff^®^qPCR SYBR^®^Green Master Mix kit (Yeasen, Shanghai, China). The reaction mixture consisted of 0.5 µL cDNA, 0.3 µL Primer-F, 0.3 µL Primer-R, 5 µL 2× Master Mix, 3.9 µL sterile water. Amplification program: 94 °C for 5 min, 94 °C for 15 s, 56 °C for 20 s, and 72 °C for 20 s. Forty cycles were performed. Gene expression levels were calculated using the 2^−∆∆Ct^ method. Each individual experiment included three biological replicates, with primers as shown in Supplementary Table S6.

### 4.5. Widely Targeted Metabolomics Assay and Statistical Analysis

To investigate the metabolic differences among J58, RX3, and RXN2, a widely targeted metabolomics assay was conducted as previously described [29]. One-month-old shoots from J58, RX3, and RXN2 were harvested, vacuum freeze-dried, and ground into a fine powder for extraction. Metabolite detection and analysis were performed using the Thermo Fisher Ultra High-Performance Liquid Chromatography Tandem Fourier Transform Mass Spectrometry (UHPLC-Q Exactive) system by Majorbio (Shanghai, China). Identified metabolites were annotated against the KEGG Compound database (version v20230830, http://www.kegg.jp/kegg/compound/, accessed on 30 August 2023), and these annotated metabolites were subsequently mapped to the KEGG Pathway database (https://www.kegg.jp/kegg/pathway.html, accessed on 30 August 2023). Each cultivar contained three biological replicates with each replicate consisting of pooled tissue from at least ten individual seedlings.

### 4.6. Data Analysis

For differences analysis, Student’s *t*-test was used when comparing two variables. A *p*-value < 0.05 was considered statistically significant.

## 5. Conclusions

In conclusion, we successfully developed a rice variety, RXN2, which is both highly productive and resistant to bacterial blight. Integrative transcriptomic and metabolomic analyses elucidated that GA and auxin contributed to plant height and grain yield, whereas JA and Prx were responsible for enhanced *Xoo* resistance in RXN2. Our research reaffirms the pivotal role of hybridization and irradiation techniques in rice breeding and offers a novel perspective for uncoupling growth and defense trade-offs. Looking ahead, the insights gained from our study on the interplay between growth and defense mechanisms in rice could potentially be translated to other major cereal crops, such as wheat and maize, to improve productivity and disease resistance. This work also paves the way for future research exploring the molecular underpinnings of growth–defense trade-offs in diverse agricultural settings.

## Data Availability

The original contributions presented in this study are included in the article/Supplementary Material. Further inquiries can be directed to the corresponding authors.

## Supplementary Materials

The following supporting information can be downloaded a: https://www.mdpi.com/article/10.3390/plants14182921/s1, Table S1. Transcriptome sequencing quality assessment; Table S2. The identified different expressed genes in J58, RX3 and RXN2 plants; Table S3. KEGG pathway analysis using the different expressed genes in RXN2 compared with J58 and RX3; Table S4. The identified different metabolites in J58, RX3 and RXN2 plants; Table S5. KEGG pathway analysis using the different metabolites in RXN2 compared with J58 and RX3; Table S6. qRT-PCR primer sequence.
